# Comparison of platelet-albumin-bilirubin (PALBI), albumin-bilirubin (ALBI), and child-pugh (CP) score for predicting of survival in advanced hcc patients receiving radiotherapy (RT)

**DOI:** 10.18632/oncotarget.25522

**Published:** 2018-06-22

**Authors:** Connie H.M. Ho, Chi-Leung Chiang, Francis A.S. Lee, Horace C.W. Choi, Jeffery C.H. Chan, Cynthia S.Y. Yeung, J.J. Huang, Mark K.H. Chan, Oliver Blanck, Frank C.S. Wong

**Affiliations:** ^1^ Department of Clinical Oncology, Tuen Mun Hospital, Hong Kong (SAR), China; ^2^ Department of Clinical Oncology, University of Hong Kong, Hong Kong (SAR), China; ^3^ University of Hong Kong-Shenzhen Hospital, Hong Kong, China; ^4^ Department of Clinical Oncology, Queen Elizabeth Hospital, Hong Kong (SAR), China; ^5^ Department of Radiation Oncology, Universitatsklinikum Schleswig-Holstein Campus Kiel, Kiel, Germany; ^6^ Department of Radiation Physics, Imperial College NHS Healthcare, Charing Cross Hospital, UK, London

**Keywords:** PALBI, radiotherapy, HCC, survival prediction

## Abstract

**Purpose:**

This work evaluated the prognostic performance of Child-Pugh (CP), albumin-bilirubin (ALBI) and platelet-albumin-bilirubin (PALBI) scores in hepatocellular carcinoma (HCC) patients undergoing radiotherapy (RT).

**Results:**

The study included 174 consecutive patients with 63% at CP A5 (*n* = 110) and 34% at CP A6 (*n* = 64). The median ALBI score was −2.39 (range: −3.61 to −1.41) with 34.5% at grade A1 (*n* = 60) and 65.5% at grade A2 (*n* = 114). The median PALBI score was −2.39 (range −3.39 to −1.24) with 33.3% at grade 1 (*n* = 58), 41.4% at grade 2 (*n* = 72) and 25.3% at grade 3 (*n* = 44). With a median follow-up of 21.7 months, the median OS of the entire cohort was 22.2 months. OS was significantly associated with the PALBI grade (*p* = 0.002) and for the ALBI grade (*p* = 0.00495), but not for the CP score (*p* = 0.46). The PALBI grade has a significantly higher AUC compared than the ALBI grade or CP scores in predicting OS. The PALBI grade was predictive of CP score decline ≥2 (20% grade 3 vs. 5.3% grade 1/2 *p* = 0.05) but the ALBI and CP scores were not.

**Conclusion:**

Among CP A HCC patients receiving radiotherapy, the PALBI and ALBI grade maybe a better prognostic tool than the CP score. The role of PALBI in predicting liver toxicity warranted further exploration.

**Methods:**

We retrospectively reviewed HCC patients treated with individualized hypo-fractionated radiotherapy (IHRT) using stereotactic technique from 2006 to 2015. We collected CP, ALBI and PALBI scores prior to treatment and analyzed their correlation with overall survival (OS) and liver toxicity.

## INTRODUCTION

Hepatocellular carcinoma (HCC) is the fifth most common cancer worldwide and ranks the second in malignancy-related mortality [1]. Patient prognosis and treatment decisions are based on tumor burden, hepatic function and performance status [2]. Thus, evaluating the patient's liver function is crucial to managing HCC.

The Child-Pugh (CP) classification incorporates five parameters: serum albumin, bilirubin, coagulation profile, ascites and encephalopathy [3–4]. The CP classification system was widely used for decades to assess patient hepatic function and has been widely adopted in the HCC staging system, management algorithm and clinical trials [5, 6]. However, the CP classification is limited by subjectivity in assessing hepatic encephalopathy and ascites, and inter-relationships between the serum albumin level and ascites [7]. In addition, the system was originally developed for patients with cirrhosis, not HCC. Therefore, the albumin-bilirubin score (ALBI) was recently proposed as a simple and objective assessment of liver function, in which the score is based solely on the serum albumin and bilirubin levels [8]. The ALBI score was developed based on an extensive international database of HCC patients. The scoring system was later validated for patients receiving resection, trans-arterial chemo-embolization (TACE), Sorafenib and stereotactic body radiation (SBRT) [9–14], and it showed potential for predicting the patient's overall survival (OS) and stratifying CP class A patients into two groups with distinct prognoses. Based on the ALBI score, the platelet-albumin-bilirubin (PALBI) score was recently developed to account for the effect of portal hypertension, with the platelet count acting as a surrogate for portal hypertension severity [15]. A recent study found that PALBI and ALBI both have a better predicting power than the CP score in HCC patients receiving aggressive therapies [16].

To date, the PALBI score has not been validated specifically in HCC patients receiving radiotherapy. Also, there were few reports to evaluate the prognostic ability of ALBI and PALBI scores in advanced HCC population receiving non-curative treatments. Our institution adopted the approach of individualized hypo-fractionated radiotherapy (IHRT) using a stereotactic technique to treat HCC patients who are ineligible for curative treatment modalities. The dose delivered was individually adapted to normal tissue dose constraints. The protocol details were previously presented in abstract form [17]. This study compared the prognostic performance of PALBI grade, ALBI grade and CP score in advanced HCC patients using our IHRT protocol. We will also evaluate their ability in predicting RT-induced liver function decline.

## RESULTS

### Patients and tumor characteristics

From 2006 to 2015, 174 consecutive patients who meet the eligibility criteria were evaluated. Baseline patient, tumor, and treatment characteristics are listed in Table 1. Of the 174 patients, 74 patients (42.5%) carried Barcelona Clinic Liver Cancer (BCLC) stage B disease and 100 (57.5%) had stage C. The median size of the tumor was 9.75 cm (range 2.3–25.7 cm). One hundred and ten patients (63.2%) had CP A5 liver function and 64 patients (36.8%) had CP A6. The median ALBI score was −2.39 (range: −3.61 to −1.41). Based on the ALBI grade, 60 patients (34.5%) were ALBI grade A1, 114 (65.5%) were grade A2. The median PALBI score was −2.39 (range −3.39 to −1.24). Fifty-eight individuals (33.3%) were PALBI grade 1, 72 (41.4%) were grade 2, and 44 (25.3%) were grade 3.

**Table 1:** Patient and tumor characteristics

Characteristics	Number of patients (%); *N* = 174
Age	
Median (Range)	62 (27−90)
Sex	
Male	149 (85.6%)
Female	25 (14.4%)
Child-Pugh Score	
A5	110 (63.2%)
A6	64 (36.8%)
Etiology^a^	
Hepatitis B	137 (78.7%)
Hepatitis C	13 (7.5%)
Alcohol	72 (41.4%)
ECOG	
0	64 (36.8%)
1	47 (27.0%)
2	63 (36.2%)
BCLC Stage	
B	74 (42.5%)
C	100 (57.5%)
Platelet count Median (Range)	188.5 (37−696)
Albumin level Median (Range)	37 (27–49)
Bilirubin level Median (Range)	11.5 (3–49)
Portal vein thrombosis	
Yes	54 (31.0%)
No	120 (69.0%)
Number of lesions	
Solitary	97 (55.7%)
Uni-nodular	37 (21.3%)
Multi-nodular	40 (23.0%)
Size of largest lesion, cm Median (Range)	9.8 (2.3−25.7)
GTV size (largest), cc Median (Range)	446.0 (9.2−4,009)
Radiation dose BED_10_ Median (Range)	37.3 (23.3–72)
Number of fractions Median (Range)	7 (5–10)

^a^allow for multiple etiology.

Table 2 shows the correlations between the CP score, ALBI and PALBI grades. Most ALBI grade A1 patients (95%) were CP A5; of the ALBI grade 2 patients, 46% were CP A5, while 54% were CP A6. There were more PALBI grade A1 patients, and more A2 patients had CP A5 liver function than CP A6 (PALBI grade A1, 83% vs 17%; PALBI grade A2, 61% vs 39%), but more PALBI grade A3 patients had CP A6 liver function (59%). The distribution of CP A5 and A6 patients were statistically different either by ALBI or PALBI grading (*p* < 0.001). Supplementary Figure 1 shows the correlations between ALBI and PALBI score.

**Table 2:** (A) Correspondences between CTP scores and ALBI grades; (B) Correspondences between CTP scores and PALBI grades

(A) ALBI grades	Child Pugh A5			Child Pugh A6	
(B) PALBI grades		Child Pugh A5	Child Pugh A6	
ALBI grade A1	57			3	
ALBI grade A2	53			61	
	Fisher's exact test			*p* < 0.001	
PALBI grade 1		48	10	
PALBI grade 2		44	28	
PALBI grade 3		18	26	
		Chi-square test	*p* < 0.001	

### Prognostic value of CP, ALBI and PALBI on survival

With a median follow-up of 21.7 months (range: 2.6–127.2 months), the median OS of the entire cohort was 22.2 months (95% confidence interval [CI]: 17.8–26.5 months). Twenty-two patients were alive at the time of analysis. Only one patient had lost follow-up at the time of analysis.

**Figure 1:**
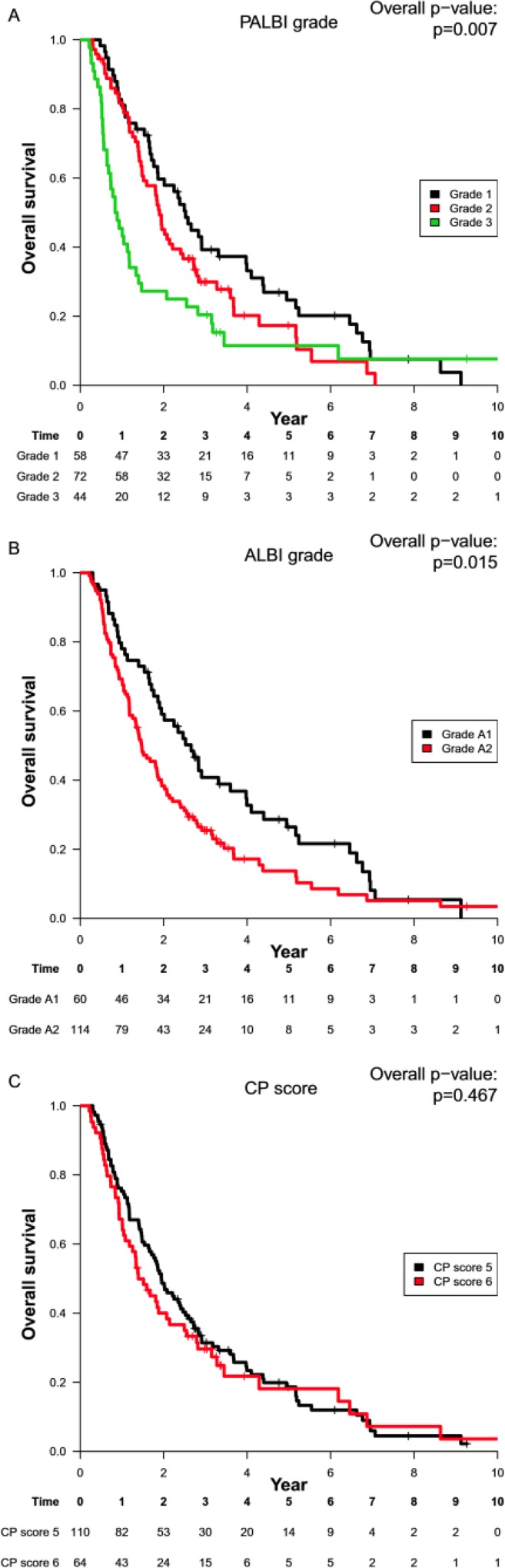
Kaplan–Meier curves of overall survival according to (**A**) PALBI grade 1 vs 2 vs 3; (**B**) ALBI grade A1 vs A2 (**C**) CP score A5 vs A6. Abbreviations: ALBI, Albumin-Bilirubin; CP, Child-Pugh; OS, overall survival; PALBI, Platelet-Albumin-Bilirubin.

For pre-treatment PALBI grade-stratified patients, the median OS was 30.3 months for grade 1 (95% CI: 22.4–47.9 months), 22.8 months for grade 2 (18.2–29.6 months), and 10.3 months for grade 3 (8.2–16.1 months). The OS stratified by PALBI grade is plotted in Figure 1A. The OS of PALBI grade 1 was statistically different from that of grade 3 (*p* = 0.010, adjusted using Holm's method) and was marginally different between grades 2 and 3 (*p* = 0.051); but was not different among grades 1 and 2 (*p* = 0.07). For pre-treatment ALBI grade-stratified patients, the OS for grades A1 and A2 was different (*p* = 0.015), with median OS was 31.9 months for grade A1 (95% CI: 22.8–47.9 months) and 17.7 months for grade A2 (95% CI: 15.1–23.2 months). The overall survival stratified by ALBI grade is plotted in Figure 1B.

For pre-treatment CP A5 and A6 patients, the median OS was 23.4 months (95% CI: 19.9–30.3 months) and 16.7 months (95% CI: 14.2–29.9 months), respectively. Figure 1C presents the OS of CP grades A5 and A6 patients which shows no statistical difference (*p* = 0.47).

**Figure 2:**
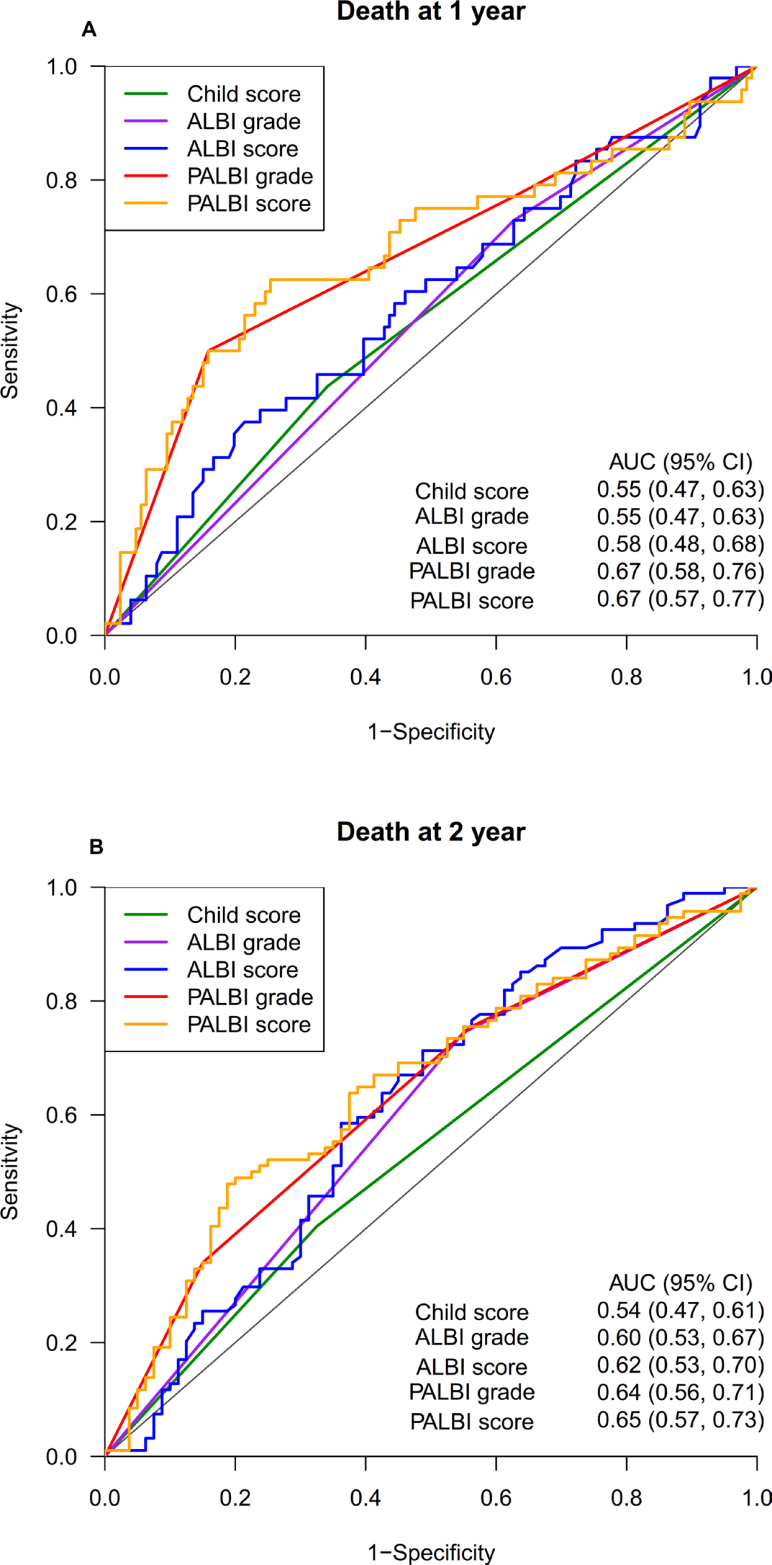
Receiver operating characteristic curves and corresponding area under the curve (AUC) for (**A**) 1-year and (**B**) 2-year OS. Abbreviations: ALBI, Albumin-Bilirubin; AUC, area under the curve; CP, Child-Pugh; CI, confidence interval; OS, overall survival; PALBI, Platelet-Albumin-Bilirubin

The discriminatory capabilities of the CP score, ALBI grade, and PALBI grade were quantified using the AUC values. The PALBI grade had higher AUC values than the ALBI grade and CP score in predicting one-year and two-year OS rates (Figure 2A and 2B). The AUC value of the PALBI grade at one year was 0.67 (95% CI: 0.58–0.76) and at two years was 0.64 (95% CI: 0.56–0.71), while the AUC value of the ALBI at 1 year was 0.55 (95% CI: 0.47–0.63) and at 2 year was 0.60 (95% CI: 0.53–0.67), and those of CP score at one year were 0.55 (95% CI: 0.47–0.63) and 0.54 (95% CI: 0.47–0.61). We also evaluated the AUC of each system using the original continuous scores. The PALBI grade had the highest AUC in predicting the one-year OS of 0.673 (95% CI: 0.57–0.77) and 2-year OS of 0.65 (95% CI: 0.57–0.73), while ALBI grade was associated with higher AUC score at the two-year interval.

### Prognostic factors of overall survival

The 1-year, 2-year OS rates were 72.3% (95% CI: 65.9–79.3%) and 45.4% (95% CI: 38.6–53.5%) respectively. In the univariate analysis of the OS, the ALBI grade, the PALBI grade, age, tumor size, the lesion numbers, performance status, presence of metastases, portal vein thrombosis, and BCLC stage were significantly associated with OS (Table 3). However, CP score was not a significant factor.

**Table 3:** Prognostic factors on overall survival by Cox's proportional hazard model

	Multivariable analysis											
	Univariable analysis			CP score			ALBI score			PALBI score		
Variables	HR	95% CI	*p*-value	HR	95% CI	*p*-value	HR	95% CI	*p*-value	HR	95% CI	*p*-value
Age (per 10 yr)	0.88	(0.77, 1.00)	0.045^*^	NS			NS			NS		
Gender Female (Ref) Male (149)	Ref 1.30	(0.81, 2.08)	0.28	NS			NS			NS		
ECOG 0 (Ref) 1 2	Ref 1.96 1.57	(1.29, 2.97) (1.05, 2.26)	0.005^**^ 0.002^**^ 0.029^*^	NS			NS			NS		
Hepatitis B Yes No (Ref)	1.09 Ref	(0.73, 1.62)	0.69	NS			NS			NS		
Size of tumor	1.10	(1.06, 1.13)	<0.001^***^	1.11	(1.08, 1.15)	<0.001^***^	1.11	(1.08, 1.15)	<0.001^***^	1.10	(1.06, 1.14)	<0.001^***^
Lesion number Solitary(Ref) Uni-nodular () Multi-nodular ()	Ref 1.55 1.59	(1.02, 2.34) (1.08, 2.34)	0.025^*^ 0.038^*^ 0.020^*^	Ref 2.42 1.78	(1.56, 3.73) (1.19, 2.66)	<0.001^***^ <0.001^***^ 0.005^**^	Ref 2.28 1.78	(1.48, 3.51) (1.19, 2.65)	<0.001^***^ <0.001^***^ 0.005^**^	Ref 2.34 1.93	(1.52, 3.60) (1.29, 2.89)	<0.001^***^ <0.001^***^ 0.001^**^
Presence of metastasis No metastasis (Ref) Metastasis	Ref 2.06	(1.29, 3.29)	0.003^**^	Ref 1.86	(1.15, 3.01)	0.012^*^	Ref 2.15	(1.31, 3.55)	0.003^**^	Ref 1.92	(1.18, 3.12)	0.009^*^
BCLC B (Ref)C	Ref 1.57	(1.13, 2.20)	0.008^**^									
Portal vein thrombosis Yes No (Ref)	1.56 Ref	(1.10, 2.20)	0.012^*^	1.68 Ref	(1.17, 2.40)	0.005^**^	1.59 Ref	(1.11, 2.28)	0.011^*^	1.60 Ref	(1.11, 2.29)	0.011^*^
Radiation dose BED_10_ ≥ 40 BED_10_ < 40 (Ref)	0.55 Ref	(0.39, 0.76)	<0.001^***^	NS			NS			NS		
CP score 5 (Ref) 6	Ref 1.13	(0.81, 1.59)	0.46	Ref 1.34	(0.94, 1.91)	0.10	-			-		
ALBI score	1.41	(1.00, 1.99)	0.0495^*^	-			1.72	(1.20, 2.48)	0.004^**^	-		
PALBI score	1.85	(1.25, 2.75)	0.002^**^	-			-			1.70	(1.12, 2.60)	0.013^*^

Note: ^*^p < 0.05; ^**^p < 0.01; ^***^p < 0.001; NS: not significant.

Abbreviations: CI, confidence level; CP score, Child-pugh score; HR, hazard ratio.

From the multivariable analysis, both the ALBI score (hazard ratio, HR: 1.72, 95% CI: 1.20–2.48; *p* = 0.004) and the PALBI score (HR: 1.70, 95% CI: 1.12–2.60, *p* = 0.013) were an independent factor in predicting OS. Other significant factors include the presence of metastasis, number of lesions, tumour size, and portal vein thrombosis. Details regarding the univariable and multivariable analyses are shown in Table 3.

### Discriminatory power of CP, ALBI and PALBI on RILD and liver function decline

Eleven patients had incomplete follow-up at 3 months, from whom 6 were included in the analysis because of the occurrence of CP score decline. Further 9 patients were excluded from the analysis due to intra-hepatic progression within 3 months. Another 12 were excluded due to unable to access CP score (mainly missing INR). For the 148 evaluable patients, 13 patients (8.8%) had liver function decline.

**Figure 3:**
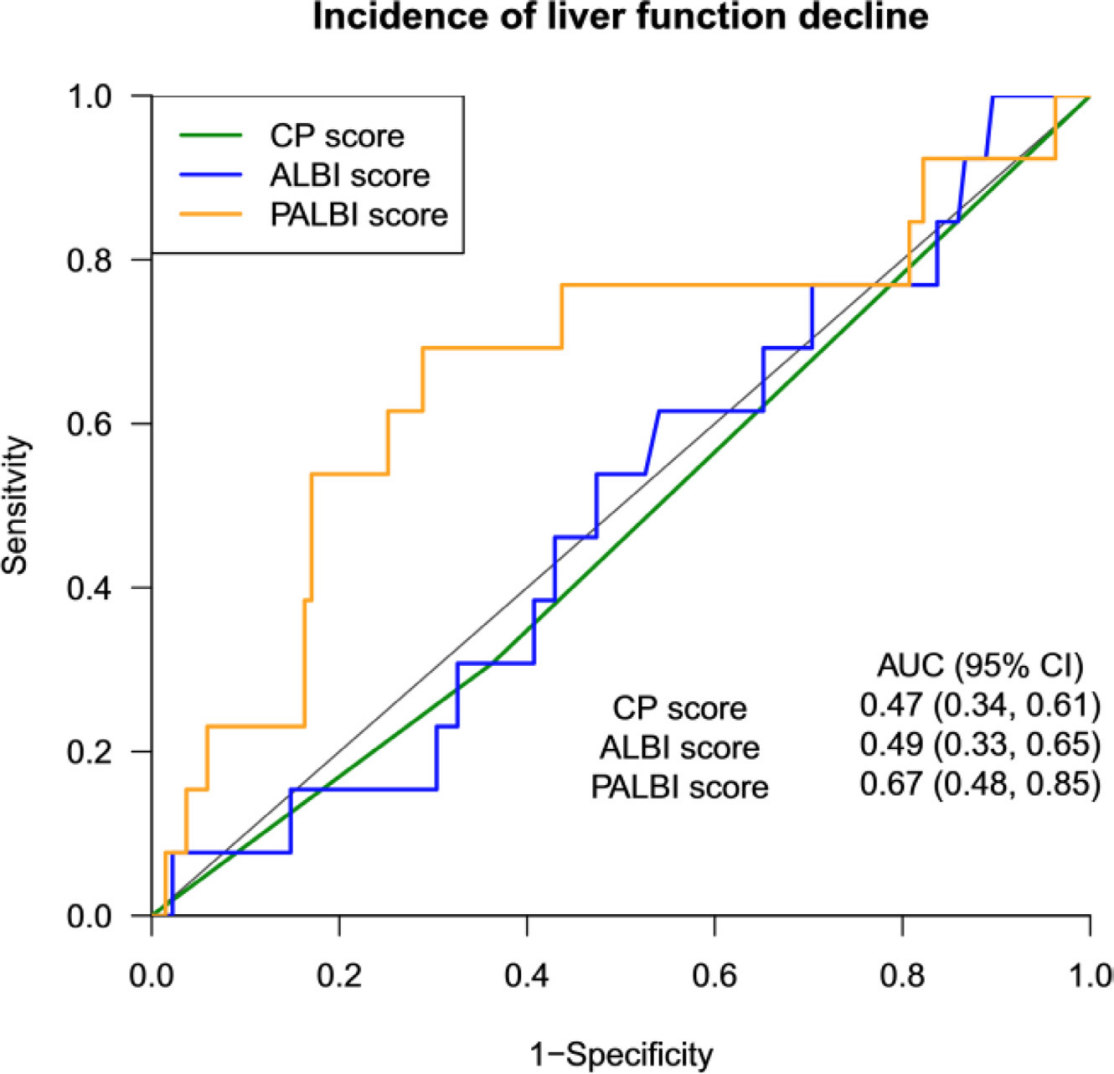
Receiver operating characteristic curves and corresponding area under the curve (AUC) for incidence of liver function decline Abbreviations: ALBI, Albumin-Bilirubin; AUC, area under the curve; CP, Child-Pugh; CI, confidence interval; PALBI, Platelet-Albumin-Bilirubin.

No patients developed classical RILD. The incidence of liver function decline rose with the pre-treatment PALBI grade with 5.7% for PALBI grade 1, 5.0% for grade 2, and 20.0% for grade 3 (*p* = 0.05). In contrast, the incidence didn't change with either the CP score (9.5% for CP5 vs. 7.5% for CP6, *p* = 0.77) or the ALBI grade (9.4% for grade 1 vs. 8.4% for grade 2, *p* = 1.0). Patients with CP decline had a trend to have a higher PALBI score (−2.18 ± 0.48 vs −2.41 ± 0.40, *p* = 0.11), while a similar trend was not observed in the ALBI score (−2.44 ± ± 0.39 vs −2.44 ± 0.43, *p* = 0.99). In terms of predicting the incidence of liver function decline (Figure 3), the PALBI score (AUC = 0.67, 95% CI 0.48–0.85) was superior than the ALBI score (AUC = 0.49, 0.33–0.65, *p <* 0.001) and the CP score (AUC = 0.47, 0.34–0.61, *p* = 0.076). The optimal cut-off value by the PALBI score was −2.23 with a sensitivity and specificity of 0.71 and at 0.69, respectively. The distribution of CP decline according to PALBI grade, ALBI grade, and CP score is shown in Table 4. The distribution of liver function decline according to PALBI score and CP score is shown in Supplementary Table 1. Dosimetric factors were not associated with the development of CP score decline ≥ 2 (Supplementary Table 2).

**Table 4:** Proportion of the incidence of liver function decline by CP score, pre-treatment ALBI grade and pre-treatment PALBI grade among 148 eligible patients

	*n*	Number of patients with liver function decline	*p*-value
CP score			0.77
5	95	9 (9.5%)	
6	53	4 (7.5%)	
ALBI grade			1.0
1	53	5 (9.4%)	
2	95	8 (8.4%)	
PALBI grade			0.051
1	53	3 (5.7%)	
2	60	3 (5.0%)	
3	35	7 (20%)	

## DISCUSSION

In the present study, we demonstrated that both PALBI and ALBI have a better prognostic ability over CP score in HCC patients treated with radiotherapy, and PALBI is superior among the classifications in predicting liver function decline.

Most HCC patients allowed to receive radiation had a classical well-preserved liver function (CP-A), as a poor liver function is associated with increased treatment-related toxicity and inferior survival [14, 18], Based on the current study, yet, not all CP-A patients have favorable prognosis according to the ALBI and PALBI grade. In agreement with the work by Lo *et al*., the ALBI grade can divide CP-A individuals receiving RT into two distinct survival cohorts [14]. Further, we demonstrated that the PALBI grade could separate CP-A into three prognostic groups. ALBI and PALBI grade 1 patients have the better survival outcome than those with grades 2 or 3. Similar trends were seen in both BCLC subgroups. On the contrary, the prognostic value of the CP score within CP-A was less well defined, and previous studies have shown conflicting results [10, 19]. In our series, there was no significant difference in survival between the CP A5 and CP A6 patients.

Our study has demonstrated that the PALBI grade has superior discriminatory power over the ALBI grade and CP score in predicting 1-year and 2-year OS. It was consistent with the findings in the study by Liu *et al*., showing that the PALBI grade out-performed other prognostic systems in HCC patients receiving aggressive therapies [16]. For a more detailed examination the prognostic ability of the ALBI and PALBI grades, it was found that a more balanced distribution of prognostic groups in PALBI grade (33.3% grade 1, 41.3% grade 2, and 26.4% grade 3) compared to the skewed one in ALBI grade (34.5% grade 1, 65.5% grade 2, and 0% grade 3). Also, among 64 CP A6 patients, majority of them were ALBI grade 2 (*n* = 61, 95%); a similar trend was also previously described by Lo *et al.* (9% grade 1, 91% grade 2) [14]. We argue that the distinctive feature of ALBI in separating CP-A into two survival groups mostly came from dividing CP A5, but not CP A6 individuals. In contrast, PALBI grade had the uniform ability in assigning patients' prognoses in both CP A5 and CP A6 (see Table 2). All the above findings suggested that, among patients with preserved liver function (CP-A), PALBI maybe a better prognostic system than ALBI and CP score.

In present study, around 25% (44/174) of CP-A individuals belongs to PALBI grade 3, which is slightly more than that of 10% in the previous one [16]. There is a couple of possible explanations. First of all, liver function of HCC patients is a complex interplay between underlying hepatic reserve and tumor burden. In contrast to the work by Liu *et al.*, [16] our cohort represents a more advanced HCC populations that majority of them are unsuitable for curative interventions, thus they are more prone to have subtle liver function decline than those with earlier stage disease. Another possibility is that more patients in present series have co-existing portal hypertension, however we don't have the detailed information in this aspect to proof our hypothesis; also, the difference in patient number between two series may also attribute to the variability.

Based on our result, we would not advocate excluding patients with PALBI grade 3 from aggressive therapy, as it could abandon a significant proportion of patients who might otherwise tolerate the treatment given the low positive predictive value of 20% when using PALBI grade 3 as cutoff. Rather, given the low toxicity rate in patients with PALBI grade 1–2 and their promising prognosis, re-irradiation or dose-escalation may be considered.

The baseline liver function is a well-recognized important factor in predicting hepatic toxicity after RT and previous studies have demonstrated that both CP and ALBI score can serve this purpose where worse CP or ALBI score was associated with increased risk of liver toxicity [14, 18, 20]. In the work by Velec *et al*., among 101 CP-A patients, CP A6 individuals had increased risk of liver toxicity (odd ratio ~5) compared to CP A5 [18] and Lo *et al*. found that the ALBI score was independently associated with hepatic function decline [14]. However, similar findings were not observed in present series, in which both CP score and ALBI grade was not predictive of CP decline ≥2. A possible explanation is that our sample number has inadequate power to detect the differences. Yet, an alternative possibility is that the CP and ALBI score is an insensitive predictor in patients with preserved liver function.

Intriguingly, in our study higher PALBI grades increased the risk of hepatic function decline. A PALBI grade 3 had a higher incidence of CP score decline ≥2 post-RT than PALBI grades 1 or 2 (20% vs. 5.3%, *p* = 0.05); PALBI was found to have superior predictive power over CP and ALBI on liver toxicity, as reflected by higher AUC value (PALBI 0.67 vs. ALBI 0.49 vs. CP 0.47, *p <* 0.05). Our results indicated that the PALBI grading, with the addition of platelet count on top of ALBI grading, might potentially serve as an objective predictor of RT-induced liver toxicity. This finding was unsurprising for several reasons. Firstly, the independent role of platelet count as a predictor of post-SBRT hepatic injury was published before [18]. Also, platelet count has been widely used in surgical setting to predict post-operative morbidity [21] Further, baseline liver function is the well known foremost factor in forecasting RT-induced liver toxicity. Better performance in assessing pre-treatment hepatic function by PALBI over ALBI, as shown in previous study and our series, may also account for its better prediction ability [16].

However, probably owing to the modest dose of radiation (median 2Gy equivalent dose in a/b ration of 10 EQD2_10_ = 37.3 Gy) prescribed, there was a low incidence of post-RT hepatic toxicity observed in our cohort (*n* = 13, 8.3% decline in CP score ≥2). Evidence has suggested the low number of events would negatively affect the validity of logistic regression model and lead to erroneous conclusion [22], thus it was impossible for us to perform multivariate analysis to control the effect of potential confounders. Also, the missing data of liver toxicity assessment have further limited the robustness of our findings. Further analysis in the large prospective database is warranted to validate our observations.

To the best of our knowledge, our series was the first to compare prognostic ability and their power in predicting liver toxicity between the PALBI, ALBI, and CP classifications in HCC patients receiving RT. Our results justified the further exploration of the PALBI grade as both survival and liver toxicity predictors. Other strengths include the relatively long follow-up period, modestly large sample size, and complete patient record. Additionally, our patients carried more advanced disease than those in similar studies; it reflected the prognostic ability of PALBI and ALBI are not limited to patients receiving radical treatment, but also in those with less favorable prognosis.

However, some limitations are inherited in the present study due to its retrospective nature. Although efforts were taken to reduce the potential biases: the confounding factors were adjusted with multivariate analysis, consecutive patients were included to minimize the selection bias, and overall survival was chosen to be the primary endpoint to reduce the bias introduced by subjective clinical interpretation, the findings from retrospective data could be at best hypothesis generating. Also, as the IHRT protocol used in our center is not a common practice around the world, the validity of this result may be limited elsewhere. Another weakness of the present study is that we lack cross-validation. Indeed, a substantial proportion of our patients were hepatitis B carriers. Therefore, further validations in a prospective setting, in particular among the non-endemic population, are required before ALBI and PALBI score can become part of the routine practice. Furthermore, only CP-A patients were included in the present analysis. As a result, there were no patients belonged to ALBI grade 3 which may affect the generalizability of results in those with poor hepatic function.

In conclusion, our study suggested, among patients with classical well-preserved liver function (CP-A), both the PALBI and ALBI grades could provide a more accurate estimation of survival than the CP score in patients receiving RT. The PALBI grade has promising potential in the prediction of liver toxicity. Despite several limitations existing in the current study, further prospective studies are justified. Future research will also need to determine whether the PALBI grade is a better prognostic and liver toxicity prediction tool.

## MATERIALS AND METHODS

### Patients

The HCC diagnosis was established either by biopsy or by the American Association for the Study of Liver Diseases (AASLD) criteria with characteristic enhancement on two imaging modalities in the presence of cirrhosis. From 2008 to 2015, 174 patients who were treated under the institutional IHRT protocol were included in this retrospective analysis, which was approved by the institutional ethics committee. The IHRT eligibility criteria was as follows: patients unsuitable for resection, liver transplantation, local ablation therapies, a minimum of 700 mL of uninvolved liver, an Eastern Cooperative Oncology Group (EGOC) performance score ≤ 2; a CP liver score of A5 to A6; and adequate organ function, defined as absolute neutrophil count (ANC) ≥ 1.5 × 10^9^ /L, creatinine ≥1.5 ×ULN, ALT or AST < 2.5 × ULN, and international normalized ratio (INR) <1.7. Extra-hepatic diseases were allowed, provided the greatest disease burden was hepatic. Patients with diffusely infiltrative disease or more than five tumor nodules were considered to be ineligible. There was no limit on tumor size.

### Treatment and follow-up

Tumor size and stage, uninvolved liver volume and organs at risk (OAR) were assessed by contrast enhanced computed tomography (CT). Patients were immobilized with vaclock plus, an in-house body frame with an abdominal compressor for motion management. Four-dimensional CT (4DCT) was acquired by means of a bellows-belt (Philips Medical Systems, Cleveland, OH, USA) placed over the patients' abdomen, which serves as a surrogate for 4DCT binning. The 4DCT dataset was sorted into ten respiratory phases, and the phase that corresponds to the mid-ventilation (mean) was chosen as the planning CT (PLCT). Delineation of the gross tumor volume (GTV) was aided by dual-phase contrast CT. From the liver and tumor motions, the planning target volume (PTV) was generated using the Van Herk margin recipe [23]. Treatment was planned with either a 6 MV or 10 MV photon based on the tumor size and location.

Regarding the dose and fractionation, our IHRT protocol divides patients into a favourable and an unfavourable group. Individuals who meet all the below criteria are classified into the favourable group: tumor size ≤ 10 cm, ECOG 0–1, and liver volume minus GTV ≥ 700 ml. For 47 favourable patients, 5–9 Gy for six fractions was prescribed; for the other 127 classified into the unfavourable group, 4 Gy for 5 to 10 fractions was prescribed. The dose was individualized by normal tissue constraints (See Table 5 for details). The normal liver was allowed to receive a biological effective dose with α/β-ratio of 3 Gy (BED 3 Gy) of 30 Gy_3_ < 40% and mean dose < 28 Gy_3_. Minor dose constraint violation in patients who were not hepatitis B (HBV) or hepatitis C (HCV) carriers and without evidence of cirrhosis was allowed.

**Table 5:** Dose constraints of the individualised hypo-fractionated radiotherapy (IHRT) protocol

	Favourable group (5–9 Gy × 6 fractions)	Unfavourable group (4 Gy× 5–10 fractions)
Small bowel or stomach	5 Gy × 6	4 Gy × 8
Large bowel	5.5 Gy × 6	4 Gy × 9
Oesophagus	6 Gy × 6	4 Gy × 10
Gall Bladder	6 Gy × 6	4 Gy × 10
Heart	6 Gy × 6	4 Gy × 10
Rib	8 Gy × 6	4 Gy × 10
Skin	7 Gy × 6	4Gy × 10

Patients were assessed every week during IHRT, once after completing treatment at six weeks, every three months for the first two years and every four months thereafter. Physical examinations and blood work were performed at every follow-up. A Tri-phasic liver CT was obtained at three months after SBRT and then every three months in the first year and every six months after that. Tumor response was measured using Response Evaluation Criteria In Solid Tumors (RECIST) criteria version 1.1.

OS was calculated from the start of radiotherapy until the date of final follow-up or death. Liver function data were obtained before IHRT at baseline within one week prior to treatment.

Classic radiation induced liver disease (RILD) was defined as an anicteric elevation in alkaline phosphatase of at least twice the upper normal limit and nonmalignant ascites within fours months after the completion of RT. Liver function decline was defined by worsening of CP score ≥ 2 after three months of completion of RT. Liver toxicity was censored at the time of intrahepatic progression or liver-directed therapies. The relevant analysis included only patients with: (1) adequate follow-up (three months for liver function decline measured by a worsening of CTP score ≥ 2 and four months for RILD) or (2) death and development of toxicity prior to the corresponding period.

### CP, PALBI and ALBI score calculation

The CP score was calculated using the total bilirubin, albumin, INR, and severity of ascites and hepatic encephalopathy. Encephalopathy was graded as absent, minimal (grade 1–2), or advanced (grade 3–4). The ALBI score was calculated using the following formula:

ALBI score = (log_10_ bilirubin × 0.66) + (−0.085 × albumin), where bilirubin is in μmol/L and albumin in g/L. The ALBI score was defined as [8]:

ALBI grade 1 (≤−2.60)

ALBI grade 2 (>−2.60 to ≤−1.39)

ALBI grade 3 (>−1.39)

The PALBI score was calculated by the following equation [15]:

PALBI = 2.02 × log_10_ bilirubin − 0.37 × (log_10_ bilirubin)^2^ − 0.04 × albumin −3.48 × log_10_ platelets + 1.01 × (log_10_ platelets)^2^. The PALBI grade was defined as

PALBI grade 1 (score ≤−2.53);

PALBI grade 2 (score>−2.53 and ≤−2.09);

PALBI grade 3 (score>−2.09).

### Statistical analysis

Distribution of patients by CP, ALBI and PALBI grades were compared by the Fisher's exact and Chi-square tests. The OS was calculated based on Kaplan–Meier estimate. The Log-rank test was used to compare outcomes among the Kaplan–Meier survival curves for potential prognostic factors. In the multivariable Cox proportional hazards regression model, the CP, ALBI and PALBI scores were included in three separate models with backward elimination used to select the remaining significant factors. The area under the receiver operating characteristic curve (AUC), equivalent to concordance index, was calculated to test the discriminatory powers for predicting one year and two year mortality rates. *P* values for multiple comparisons were adjusted using Holm's method. *P <* 0.05 was considered as statistically significant. R version 3.2.5 (Vienna, Austria) was used for statistical analysis.

## SUPPLEMENTARY MATERIALS FIGURE AND TABLES


