# Supplementary material for: Comparison of platelet-albumin-bilirubin (PALBI), albumin-bilirubin (ALBI), and child-pugh (CP) score for predicting of survival in advanced hcc patients receiving radiotherapy (RT)
Source: Oncotarget. 2018 Jun 22;9(48):28818–29. doi: 10.18632/oncotarget.25522 (PMC6034750; doi:10.18632/oncotarget.25522)
Supplement: Supplementary file 1 [file oncotarget-09-28818-s001.pdf]

Comparison of platelet-albumin-bilirubin (PALBI), albumin-bilirubin (ALBI), and child-pugh (CP) score for predicting of survival in advanced hcc patients receiving radiotherapy (RT)

SUPPLEMENTARY MATERIALS

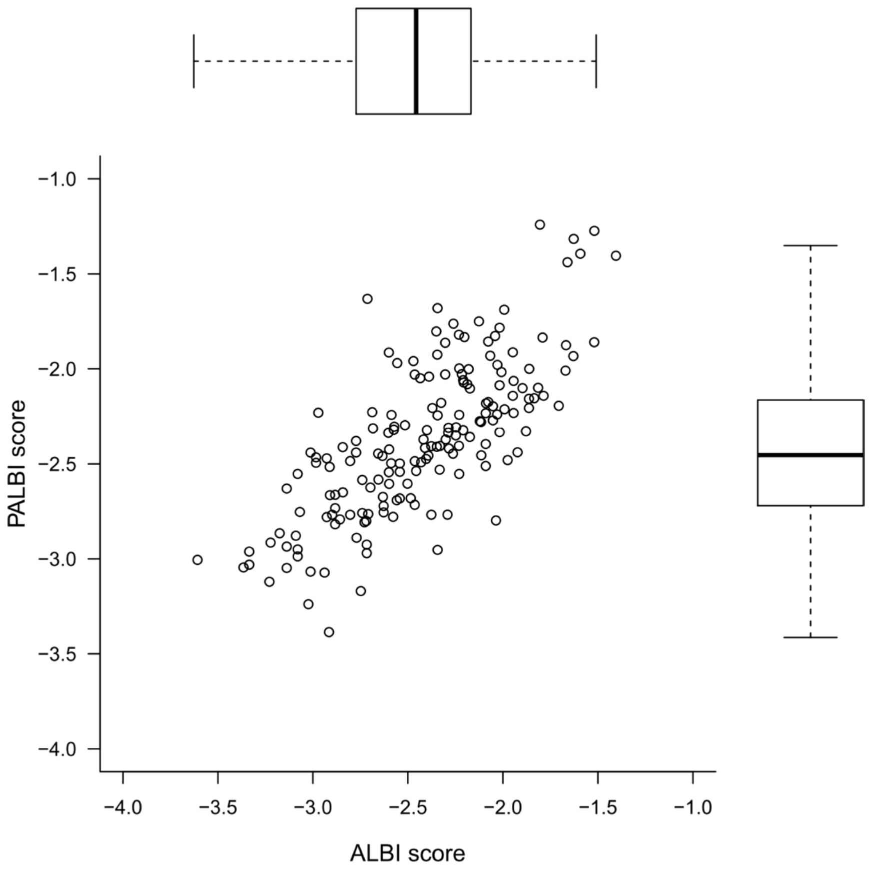

Supplementary Figure 1: Correlation between albumin-bilirubin (ALBI) score and platelet-albumin-bilirubin (PALBI) score (Spearman correlation = 0.746, *p* < 0.001). Abbreviations: ALBI, Albumin-Bilirubin; PALBI, Platelet-Albumin-Bilirubin.

Supplementary Table 1: Distribution of CP score decline ≥ 2 post-RT according to PALBI score and CP score

	PALBI < −2.23 (*n* = 48)	PALBI score ≥ −2.23 (*n* = 100)	Total
CP A5	6/22 (27.3%)	3/73 (4.1%)	95 (9.5%)
CP A6	3/26 (11.5%)	1/27 (3.7%)	53 (7.5%)

Supplementary Table 2: Dosimetric analysis in assessing the risk of liver toxicity

	CP decline < 2 Mean ± SD	CP decline ≥ 2 Mean ± SD	*P* value
Liver (cc)	1811 ± 881	2164 ± 695	0.11
Liver-GTV (cc)	1184 ± 353	1403 ± 389	0.08
V30 (Gy)	35.4 ± 46.4	35.3 ± 7.4	0.99
Mean liver dose (Gy)	21.6 ± 6.4	23.8 ± 3.5	0.07

Abbreviations: CP, Child-Pugh; CI, confidence interval; PALBI, Platelet-Albumin-Bilirubin; SD, standard deviation.
